# Effect of Glucagon-Like Peptide-1 Receptor Agonists on Renal and Cardiovascular Risk Factors in Patients With Type 2 Diabetes Mellitus: A Retrospective Study

**DOI:** 10.1155/jdr/2663671

**Published:** 2025-11-04

**Authors:** Daniel Yuan, Venkat N. Vangaveti, Oluwatosin A. Arojojoye, Usman H. Malabu

**Affiliations:** ^1^Translational Research in Endocrinology and Diabetes (TREAD), College of Medicine and Dentistry, James Cook University, Townsville, Queensland, Australia; ^2^Townsville University Hospital, Townsville, Queensland, Australia

**Keywords:** Australia, cardiovascular, glucagon-like peptide-1 receptor agonist, North Queensland, renal, Type 2 diabetes mellitus

## Abstract

**Aims:**

This research explores the impact of glucagon-like peptide-1 receptor agonist (GLP-1RA) on key risk factors associated with kidney and cardiovascular diseases in Indigenous (Aboriginal and/or Torres Strait Islander) and non-Indigenous adults living with Type 2 diabetes, receiving care at a regional health facility in North Queensland, Australia.

**Methods:**

This retrospective study included patients who attended the diabetes clinic at a regional hospital between January 2016 and January 2020. Data was extracted from electronic medical records. Basic demographic characteristics along with blood pressure, body weight, BMI, urine albumin creatinine ratio (UACR), serum creatinine, estimated glomerular filtration rate (eGFR), HbA1c, total cholesterol, low-density lipoprotein cholesterol (LDL-C), high-density lipoprotein cholesterol (HDL-C), and triglyceride levels were retrieved from initial presentation, 6 months, and 12 months post. Data was analyzed using IBM SPSS 28 with appropriate statistical tests applied.

**Results:**

The study involved a total of 164 patients. GLP-1RA use resulted in a significant reduction of HbA1c between 0 and 6 months (8.7%–7.9%, *p* < 0.01) and 0 and 12 months (8.7%–8.1%, *p* < 0.01). Significant reduction in weight between 0 and 6 months (115.9–114.0 kg, *p* < 0.001), 6 and 12 months (114.0–112.5 kg, *p* = 0.004), and 0 and 12 months (115.9–112.5 kg, *p* < 0.001) was also seen. However, there were no statistically significant differences in all measures of lipid profile and no significant changes in UACR and eGFR.

**Conclusions:**

This study affirms the effectiveness of GLP-1RAs as a glycemic control agent with an additional benefit of weight reduction across a 12-month period in adult T2DM patients. No effect on other cardiovascular parameters apart from weight or renal risk factors was observed. Further investigation into the influence of GLP-1RAs on these would be beneficial.

## 1. Introduction

Type 2 diabetes mellitus (T2DM) is a chronic condition in which the body becomes resistant to insulin, disrupting normal metabolism and leading to persistent hyperglycemia. Prolonged hyperglycemia in combination with other metabolic disorders in individuals with diabetes mellitus can result in damage to organ systems, leading to the development of fatal complications; the most common of which are microvascular and macrovascular complications which lead to elevated cardiovascular (CV) risk [1, 2]. T2DM patients have a twofold increased risk of CV complications compared with people without T2DM after accounting for traditional risk factors. CV complications are primarily responsible for mortality among people with diabetes, and those with diabetic kidney disease (DKD), a leading contributor to end-stage renal failure and a key microvascular complication of T2DM, are especially vulnerable to these complications [3]. T2DM is a primary cause of chronic kidney disease (CKD), which accounts for about half of all patients receiving renal replacement therapy (RRT) globally [4]. DKD is initiated as a result of chronic hyperglycemia that causes oxidative stress and inflammation, which affect the structure and function of the kidney, leading to impaired renal function and albuminuria. Prolonged elevation of blood glucose levels can harm the kidneys' small blood capillaries, reducing their ability to efficiently filter waste and fluids from the blood.

Glucagon-like peptide-1 receptor agonists (GLP-1RAs) represent a group of antidiabetic medications that have recently gained attention as a promising therapeutic approach for renal and CV conditions in individuals living with T2DM, which are also used in some cases to treat obesity [5].

There are different reports concerning the therapeutic effects of GLP-1RAs on CV and renal outcomes. For example, Gerstein et al. [6] reported that efpeglenatide, an exendin-based GLP-1RA, significantly reduced critical renal and CV risks in people with T2DM. Lin et al. [7] indicated that while GLP-1RAs demonstrated renoprotective effects through the reduction of composite renal outcomes among patients suffering from advanced DKD, they showed no measurable impact on composite CV events in comparison with dipeptidyl peptidase 4 inhibitors (DPP-4is). Additionally, a systematic review by Yamada et al. [8] comparing the impacts of GLP-1RAs with sodium-glucose cotransporter-2 inhibitors (SGLT2is) in those affected by T2DM and CKD concluded that SGLT2is were correlated with a reduced risk of cardiorenal events, whereas GLP-1RAs were not found to significantly reduce either outcome.

A retrospective study was conducted to explore the association of GLP-1RAs with UACR and serum creatinine levels in adult patients with T2DM undergoing treatment at a regional hospital in North Queensland, Australia. As far as we are aware, no specific studies have been conducted to examine the association between GLP-1RAs and CV and renal risk factors among individuals with diabetes in the North Queensland population. This population is unique due to its higher prevalence of T2DM, greater proportion of Indigenous people, and higher rates of obesity compared to the rest of Australia [9]. The study population included Indigenous Australians, comprising Aboriginal and Torres Strait Islander people and non-Indigenous Australians, supporting the representation of diverse population groups within Australia. We also investigated the relationship between GLP-1RAs use and risk factors for CV disease such as HbA1c, lipid profile, body mass index (BMI), and body weight.

## 2. Materials and Methods

### 2.1. Eligibility

All patients presenting at the diabetes and endocrine clinic at the Townsville University Hospital (TUH) between January 2016 and January 2020 were enlisted in the research. Individuals aged 18 years and older with a diagnosis of T2DM and taking a GLP-1RA were eligible to be included. Patients < 18 years of age, taking GLP-1RA for < 6 months, or patients with diabetes mellitus types other than Type 2 were not eligible for the study. Ethics approval was granted from the TUH and James Cook University human research ethics committees (HREC/QTHS/74855 and H8675). To ensure participant privacy, all data were anonymized prior to analysis by removing identifiable patient information. The anonymized dataset was stored on a password-protected institutional server in compliance with applicable data protection regulations.

### 2.2. Data Extraction

Data extraction involved obtaining data from two electronic systems utilized in patient care at TUH. An integrated electronic medical record (iEMR) was employed to obtain body weight, BMI, and blood pressure (systolic [SBP] and diastolic [DBP]), along with the basic characteristics of age, ethnicity, gender, and antidiabetic medications. The pathology laboratory AUSLAB system was employed to extract biochemical measurements. These included UACR, serum creatinine, eGFR, HbA1c, total serum cholesterol, LDL-C, HDL-C, and triglyceride levels. Data were collected at baseline (initial presentation to the diabetes clinic), as well as at 6- and 12-month follow-up intervals. However, data were not available for all participants at all three time points. Therefore, a complete-case approach was employed, including only participants with available data for the relevant variables at the specified time points.

### 2.3. Data Analysis

IBM SPSS Version 28 was used to analyze the data. Basic descriptive and frequency analyses of the study sample were implemented on demographic characteristics. Continuous variables were tested for normality. Nonparametric data analysis was carried out using Wilcoxon-signed rank test. Differences in renal and CV parameters between different types of GLP-1RAs were determined using analysis of covariance (ANCOVA). Chi-squared analysis was performed for investigating the association between categorical variables. Significant values of up to 0.10 in the univariate analysis or clinically important factors were entered into the binary logistic regression analysis. A significance level of *p* ≤ 0.05 was used, and Bonferroni adjustments were made for multiple testing to control for Type I error.

## 3. Results

### 3.1. Study Characteristics

Participant characteristics are presented in Table 1. Overall, a total of 164 patients were analyzed in this study. Of the included participants, 53.7% were female, and 46.3% were male. Participants had a median age of 58 (IQR 49–64). Eighty-six point six percent were of non-Indigenous ethnicity, and Aboriginal and/or Torres Strait Islander participants comprised 13.4% of the study population. Exenatide was the most used GLP-1RA, followed by dulaglutide and liraglutide. The most common concurrent antidiabetic treatment was metformin, followed by insulin and sulfonylurea medications. Other antidiabetic treatments also used were SGLT2is, DPP4 inhibitors, and pioglitazone.

### 3.2. Association Between GLP-1RA Use and Renal Parameters in Adult T2DM Patients

Renal parameters, including eGFR, UACR, serum creatinine, SBP, and DBP, were analyzed using nonparametric analysis. The results of each parameter across the different time points are presented in Table 2. A statistically meaningful difference was seen in serum creatinine levels between 0 and 6 months (73 vs. 72 *μ*mol/L, *p* = 0.05). No differences reaching statistical significance were noted in serum creatinine levels between 6 and 12 months and 0 and 12 months. Furthermore, no statistically significant changes between 0 and 6, 0 and 12, and 6 and 12 months for the parameters of UACR and eGFR were observed. Both the median UACR and eGFR values at all three time points were within the normal limits (UACR 0–3 mg/mmol and eGFR > 60 mL/min/1.73 m^2^). There were no statistically significant differences for both SBP and DBP.

### 3.3. Association Between GLP-1RA Use and CV Risk Factors in Adult T2DM Patients

**Figure 1:**
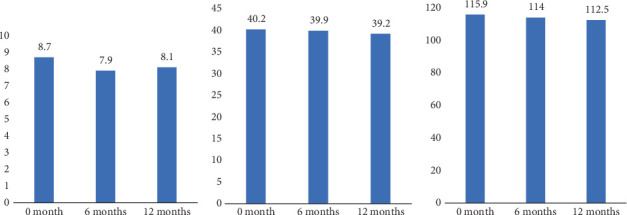
Graphical representations of (a) HbA1c, (b) BMI, and (c) weight over 12 months. HbA1c levels are expressed as percentage, BMI in kilograms per square meter, and weight in kilograms.

HbA1c, LDL-C, HDL-C, triglycerides, and total cholesterol levels along with weight and BMI were examined as CV risk factors in this study. The median HbA1c at 0 months was 8.7% (8.8–10.0), at 6 months was 7.9% (7.9–9.2), and at 12 months was 8.1% (7.1–9.3) (Figure 1a and Table 2). The differences between baseline and 6 months and baseline and 12 months were statistically significant (*p* < 0.01), while the difference during the 6- to 12-month interval was not (*p* > 0.05) (Table 2). The median BMI at baseline was 40.2 kg/m^2^ (32.3–45.3 kg/m^2^), at 6 months was 39.9 kg/m^2^ (35.1–44.2 kg/m^2^), and at 12 months was 39.2 kg/m^2^ (33.6–43.4 kg/m^2^) (Figure 1b and Table 2). The differences across all three time points reached statistical significance (*p* < 0.05, Table 2). Median body weight at 0 months was 115.9 kg (96.2–132.8 kg), at 6 months was 114.0 kg (96.0–131.0 kg), and at 12 months was 112.5 kg (94.0–125.3) (Figure 1c and Table 2). Similar to BMI, the differences across all time points were significant at the statistical level (*p* < 0.05, Table 2). No significant differences were detected for all measures of lipid profile.

### 3.4. Factors Associated With a Minimum 5% Reduction in HbA1c and Weight at 12 Months

Logistic regression (binary outcome) was performed to determine the effects of gender, age, ethnicity, type of GLP-1RA (exenatide vs. dulaglutide), metformin, sulfonylurea, DPP4 inhibitor, SGLT2i, and insulin use along with baseline and 12-month HbA1c, weight, BMI, and SBP values on the likelihood of achieving a minimum of 5% lowering of HbA1c levels 12 months postbaseline. Statistical analysis indicated that the model was significant (*χ*^2^(4) = 42.421, *p* < 0.001). The model explained 41% (Nagelkerke *R*^2^) of variance and correctly classified 74.1% of cases. Higher HbA1c at 0 months and metformin use were variables that were associated with an increased likelihood of achieving a 5% decline in HbA1c after 12 months (Table 3). The use of exenatide as opposed to dulaglutide and a higher SBP at 12 months were linked to a reduced likelihood of achieving a 5% reduction in HbA1c at 12 months. The effects of all variables in this logistic regression model are detailed in Table 3. Logistic regression analysis was also undertaken to ascertain the effects of age, gender, ethnicity, type of GLP-1RA (exenatide vs. dulaglutide), metformin, sulfonylurea, SGLT2i use, and baseline weight along with baseline and 12-month HbA1c, LDL-C, and triglyceride values on the likelihood of achieving a minimum of 5% loss in weight over 12 months. The model was statistically significant (*χ*^2^(4) = 26.517, *p* = 0.022). The model explained 45.1% (Nagelkerke *R*^2^) of variance and correctly classified 77.9% of cases. The use of sulfonylurea medications correlated with a lower probability of achieving a 5% weight loss at 12 months (OR 0.116, 95% CI: 0.015–0.907, *p* = 0.04). No other variables showed statistically significant associations (*p* > 0.05, Table 3).

## 4. Discussion

In this study, GLP-1RA exhibited decreased HbA1c levels at 6-month and 12-month follow-ups, respectively, when compared to baseline among adults suffering from T2DM from Indigenous (including Aboriginal and/or Torres Strait Islander) and non-Indigenous backgrounds. GLP-1RA was also observed to reduce both body weight and BMI across the time points. Furthermore, serum creatinine significantly decreased after 6 months of GLP-1RA use; however, no significant reduction was observed at 12 months and no significant changes in UACR and eGFR across all time points.

Our data on HbA1c are consistent with well-established literature surrounding the efficacy of GLP-1RA as a glycemic control agent in T2DM. A wealth of data from clinical trials in conjunction with meta-analyses has proven that GLP-1RAs are potent therapeutic agents in exercising glycemic control for patients with T2DM [10–13]. Recent evidence from a specialist diabetes clinic in Australia examining the influence of GLP-1RA shortages on glycemic control indicated that the unavailability of GLP-1RAs led to a significant rise in median HbA1c, further confirming its effectiveness as a hypoglycemic agent [14]. GLP-1RAs lowered HbA1c levels in patients with T2DM at 12 months compared to baseline in our study population. Furthermore, this study found that a higher baseline HbA1c resulted in a higher probability of reaching a 5% drop in HbA1c at 12 months. This finding aligns with a meta-analysis indicating that elevated baseline HbA1c levels are predictive of a stronger glycemic response to GLP-1RA treatment [15]. In contrast, Dahlqvist et al. [16] found that elevated HbA1c levels did not predict a greater reduction in HbA1c with liraglutide, a GLP-1RA. This discrepancy may be attributed to the influence of other factors such as age, diabetes duration, concomitant medications, and individual variability in response to GLP-1RA treatment.

We also observed that the use of exenatide was less likely to achieve a 5% reduction in HbA1c at 12 months relative to dulaglutide. This is likely due to variations in the mechanisms by which the two drugs act. Dulaglutide and exenatide are categorized as long-acting and short-acting GLP-1RAs, respectively, depending on the activation duration of the GLP-1 receptor [17]. Contrary to our result, a meta-analysis that evaluated the efficacy of dulaglutide in comparison to liraglutide and exenatide among patients with Type 2 diabetes observed that dulaglutide and exenatide had similar effects on changes in HbA1c [18]. However, our finding is similar to the results of Wysham et al. [19], who observed that dulaglutide provided better glycemic control relative to exenatide. Likewise, according to a retrospective real-world study at a single center, dulaglutide demonstrated a significantly greater hypoglycemic effect than exenatide in T2DM patients [10].

In addition to observing a reduction in HbA1c, we observed a reduction in both weight and BMI values throughout the study time points. This result demonstrates the relationship between GLP-1RAs and reduction in body weight in our study population. Weight loss and reductions in BMI are related to improved clinical outcomes in T2DM patients, including better glucose regulation, and decreased risk of CV events, and in some cases, diabetes remission [20, 21]. A 5% reduction in total percent fat has demonstrated efficacy in lowering HbA1c and decreasing reliance on insulin therapy [22].

We also observed that the use of sulfonylurea medications resulted in a lower likelihood of achieving a 5% weight reduction at 12 months. A well-known adverse effect of these agents is weight gain, and this could have countered the weight loss brought about by GLP-1RAs [23].

Not only are excess weight and obesity strong risk factors for T2DM, but they also increase the risk of major T2DM-related complications and overall mortality [24, 25]. Weight loss is one of the most common initial management strategies offered to patients with T2DM. The efficacy of GLP-1RAs on weight loss in patients with T2DM makes these agents a highly attractive option that may potentially be implemented earlier in the management of T2DM. Furthermore, given the link between obesity and CV disease, the weight loss and HbA1c reduction achieved with GLP-1RAs may help explain their observed CV benefits.

Evidence suggests that GLP-1RAs can suppress oxidative stress, fibrotic changes, and apoptotic activity in the kidneys [26–28]. The results from these experimental studies have led to significant interest in the potential of renal protection that may be offered by GLP-1RAs in patients with T2DM. GLP-1RAs have been shown to reduce the risk of composite renal outcomes by 17%, with the primary contribution arising from a 24% reduction in macroalbuminuria [29, 30]. In our recent systematic review and meta-analysis, it was observed that patients with T2DM taking GLP-1RAs can achieve a 16.14% reduction in UACR [31]. However, despite these previous results, this present study showed no significant statistical change in UACR or eGFR. This may, in part, be attributed to the fact that the median values for both renal parameters at all time points were in a clinically normal range (UACR between 0 and 3 mg/mmol and eGFR > 60 mL/min/1.73 m^2^). Although the reduction in serum creatinine between 0 and 6 months was statistically significant, this finding itself is unlikely to translate into clinical outcomes. Firstly, the absolute difference of 1 *μ*mol/L in serum creatinine between 0 and 6 months is minimal and unlikely to be clinically meaningful, as creatinine levels can be influenced by various factors. Moreover, serum creatinine levels at all time points remain within clinically acceptable ranges; therefore, despite the reduction, they are still considered clinically normal. This outcome is consistent with findings from a meta-analytic review of randomized controlled trials, which showed that GLP-1RA therapy had no effect on serum creatinine levels [32]. However, future prospective studies investigating a larger population with a known duration of T2DM may serve to assist clinicians in determining the efficacy of GLP-1RAs on these renal parameters.

Our study has some limitations despite its findings. First, it was retrospective in nature, with a relatively short study period and small sample size. This creates challenges when comparing data across time points, as missing data can occur. While certain statistical methods can minimize these effects, they cannot fully restore the original statistical power. The relatively small number of participants also limits the ability to capture a diverse range of patients with both early and late complications of T2DM. Additionally, the short duration of the study restricts the ability to observe the development or prevention of complications, given the chronic nature of the disease.

Furthermore, subgroup analyses were constrained by limited sample sizes, which may have limited the statistical sensitivity to detect significant changes. As such, findings from these analyses should be interpreted with caution. The logistic regression model may also be subject to overfitting, as the number of predictors included was relatively high compared to the number of events. Although statistical corrections were applied, stricter model selection methods such as stepwise regression or shrinkage techniques (e.g., LASSO) could provide more robust and generalizable results and should be considered in future analyses.

Lastly, our study focused only on risk factors for CV and renal complications of T2DM and did not examine clinical endpoints such as the occurrence of serious CV complications and advancement to RRT.

It is worth mentioning that Aboriginal and/or Torres Strait Islander individuals were represented in the study population. Although subgroup analyses by Indigenous status were not performed due to sample size constraints, their inclusion is important for ensuring that the findings reflect the diversity of the Australian population and support more inclusive research practices.

It is equally important to recognize that, while the examined risk factors are well-established predictors of CV and renal disease in T2DM, they do not replace clinical judgment. In this study, although significant reductions in weight and BMI were seen, no significant reductions in lipid profiles were observed. The lipid profile results are consistent with a recent retrospective study investigating the long-term effect of GLP-1RA use on lipid levels and HbA1c in T2DM, which found no direct association between lipid profile and 6 months of GLP-1RA treatment [33]. Additionally, a network meta-analysis and systematic review of GLP-1RAs in people with T2DM suggests that they exert a limited effect on certain lipid markers [34]. However, contrary to our results, Aoki et al. [35] observed in a pilot study that the administration of liraglutide (a newer generation of GLP-1RAs) decreased low-density lipoprotein cholesterol and total cholesterol in Japanese T2DM patients, with no changes in markers of cholesterol synthesis and absorption. The missing data on other non-antidiabetic medication patients were taking could have explained this result. Nevertheless, it is also important to consider the relatively small absolute magnitude in reduction of weight and BMI which although statistically significant may not necessarily translate into more significant clinical outcomes. This situation highlights the need for clinicians to take the results of this study in conjunction with individual patient circumstances to make an appropriate informed decision. Finally, while GLP-1RAs were examined as a class, there was no data on newer GLP-1RAs such as liraglutide and semaglutide.

## 5. Conclusion

In conclusion, this retrospective study validates the effectiveness of GLP-1RAs as a glycemic control agent with an additional benefit of weight loss across a 12-month period in adult Indigenous and non-Indigenous Australians living with T2DM. No clinically relevant associations between the use of GLP-1RAs and renal/CV risk factors were observed in this study.

## 6. Recommendations

Given the increasing popularity of newer GLP-1RAs over more traditional agents like exenatide, future studies examining whether these agents show similar efficacy will be useful to better elucidate the exact role that GLP-1RAs play in the management of T2DM, particularly in the delay of the progression of DKD and T2DM-associated CV disease. As there was no prospective control group in our study, we suggest that future prospective, randomized studies be conducted to better establish the effects of GLP-1RAs in this population.

## Figures and Tables

**Table 1:** Demographic characteristics of included participants.

**Characteristic**	**Number**
Gender (*n*, %)	
Male	76 (46.3)
Female	88 (53.7)
Age (median, IQR)	58 (49–64)
Ethnicity (*n*, %)	
Aboriginal and/or Torres Strait Islander	22 (13.4)
Non-Aboriginal and/or Torres Strait Islander	142 (86.6)
Type of GLP-1RA used (*n*, %)	
Exenatide	117 (71.3)
Dulaglutide	58 (35.4)
Liraglutide	4 (2.3)
Concurrent antidiabetic treatment used (*n*, %)	
Metformin	143 (87.2)
Insulin	95 (57.9)
Sulfonylurea	51 (31.1)
SGLT2 inhibitor	47 (28.7)
DPP4 inhibitor	14 (8.5)
Pioglitazone	2 (1.2)

Abbreviations: DPP4, dipeptidyl-peptidase 4; GLP-1RA, glucagon-like peptide-1 receptor agonist; IQR, interquartile range; *n*, number of participants; SGLT2, sodium-glucose cotransporter 2.

**Table 2:** Renal and cardiovascular parameters at baseline, 6 months, and 12 months.

**Parameter**	**n** **, 0 mths**	**Median (IQR), 0 mths**	**n** **, 6 mths**	**Median (IQR), 6 mths**	**n** **, 12 mths**	**Median (IQR), 12 mths**	**p** ** values**
UACR (mg/mmol)	137	1.7 (1.0–4.8)	90	1.7 (0.9–4.6)	93	1.6 (1.6–4.0)	0–6 mths: 0.904 0–12 mths: 0.153 6–12 mths: 0.965
eGFR (mL/min/1.73 m^2^)	160	90 (71–90)	137	90 (73–90)	128	90 (70–90)	0–6 mths: 0.114 0–12 mths: 0.225 6–12 mths: 0.614
Creatinine (*μ*mol/L)	153	73 (62.3–87)	133	72 (60.0–84.5)	122	71.5 (63.8–90.3)	0–6 mths: 0.05 0–12 mths: 0.779 6–12 mths: 0.47
Systolic blood pressure (mmHg)	162	132 (121–143)	141	132 (121–143.5)	131	133 (121–142)	0–6 mths: 0.938 0–12 mths: 0.314 6–12 mths: 0.537
Diastolic blood pressure (mmHg)	162	77 (72–84)	141	77 (72–82.5)	131	76 (72–81)	0–6 mths: 0.45 0–12 mths: 0.611 6–12 mths: 0.456
HbA1c (%)	164	8.7 (8.8–10.0)	139	7.9 (7.9–9.2)	137	8.1 (7.1–9.25)	0–6 mths: < 0.01 0–12 mths: < 0.01 6–12 mths: 0.794
LDL-C (mmol/L)	121	2.1 (1.47–2.8)	90	2.0 (1.4–2.9)	93	2.1 (1.4–3.0)	0–6 mths: 0.308 0–12 mths: 0.219 6–12 mths: 0.227
HDL-C (mmol/L)	126	1.0 (0.87–1.0)	93	1.0 (0.85–1.2)	92	1.0 (0.88–1.2)	0–6 mths: 0.851 0–12 mths: 0.394 6–12 mths: 0.877
Total cholesterol (mmol/L)	134	4.1 (3.5–4.9)	98	4.2 (3.5–5.0)	97	4.1 (3.3–5.1)	0–6 mths: 0.087 0–12 mths: 0.409 6–12 mths: 0.271
Triglycerides (mmol/L)	132	2.2 (1.6–3.0)	98	2.0 (1.5–2.9)	95	1.9 (1.4–2.9)	0–6 mths: 0.099 0–12 mths: 0.08 6–12 mths: 0.402
Weight (kg)	162	115.9 (96.2–132.8)	147	114.0 (96.0–131)	134	112.5 (94.0–125.3)	0–6 mths: < 0.001 0–12 mths: < 0.001 6–12 mths: 0.004
BMI (kg/m^2^)	162	40.2 (32.3–45.3)	147	39.9 (35.1–44.2)	134	39.2 (33.6–43.4)	0–6 mths: < 0.001 0–12 mths: < 0.001 6–12 mths: 0.003

Abbreviations: BMI, body mass index; eGFR, estimated glomerular filtration rate; HbA1c, hemoglobin A1c; HDL-C, high-density lipoprotein cholesterol; IQR, interquartile range; LDL-C, low-density lipoprotein cholesterol; mths, months; *n*, number of participants; UACR, urine albumin creatinine ratio.

**Table 3:** Variables tested by logistic regression for a minimum 5% reduction in HbA1c or weight at 12 months.

	**Unadjusted OR (95% CI)**	**p** ** value**	**Adjusted OR**	**p** ** value**
Variables associated with a minimum 5% reduction in HbA1c at 12 months				
Age	0.985 (0.954–1.017)	0.985	1.015 (0.967–1.066)	0.546
Male gender	0.875 (0.645–1.188)	0.388	0.338 (0.079–1.435)	0.141
Non-Indigenous ethnicity	1.045 (0.634–1.721)	0.860	0.944 (0.190–4.688)	0.944
Exenatide vs. Dulaglutide	0.758 (0.553–1.039)	0.111	0.218 (0.061–0.778)	**0.019**
Metformin use	1.273 (0.765–2.117)	0.308	4.713 (1.027–21.62)	**0.046**
Insulin use	1.110 (0.807–1.527)	0.513	1.206 (0.402–3.611)	0.738
Sulfonylurea use	0.652 (0.437–0.971)	0.019	0.500 (0.156–1.605)	0.244
DPP4 inhibitor use	0.706 (0.325–1.530)	0.306	0.134 (0.009–2.020)	0.147
SGLT2 inhibitor use	1.052 (0.762–1.453)	0.759	1.085 (0.334–3.531)	0.244
HbA1c at 0 months	1.495 (1.172–1.908)	0.001	1.884 (1.279–2.776)	**0.001**
Weight at 0 months	1.000 (0.988–1.012)	0.979	0.764 (0.491–1.188)	0.231
SBP at 0 months	1.012 (0.991–1.033)	0.278	1.010 (0.979–1.042)	0.540
BMI at 0 months	1.002 (0.963–1.041)	0.939	2.894 (0.773–10.83)	0.115
Weight at 12 months	1.001 (0.988–1.014)	0.938	1.371 (0.863–2.178)	0.181
SBP at 12 months	0.986 (0.964–1.009)	0.229	0.962 (0.928–0.996)	**0.030**
BMI at 12 months	1.000 (0.959–1.043)	0.994	0.300 (0.075–1.195)	0.088
Variables associated with a minimum 5% reduction in weight at 12 months				
Age	0.976 (0.944–1.010)	0.159	0.926 (0.850–1.008)	0.075
Male gender	0.753 (0.456–1.246)	0.263	0.465 (0.087–2.488)	0.371
Non-Indigenous ethnicity	0.739 (0.382–1.429)	0.399	0.062 (0.004–1.066)	0.055
Exenatide vs. Dulaglutide	1.411 (0.697–2.858)	0.317	1.863 (0.339–10.25)	0.475
Metformin use	0.562 (0.337–0.937)	0.047	0.153 (0.017–1.388)	0.095
Sulfonylurea use	0.582 (0.317–1.068)	0.063	0.116 (0.015–0.907)	**0.040**
SGLT2 inhibitor use	0.851 (0.490–1.478)	0.559	0.521 (0.106–2.558)	0.422
HbA1c at 0 months	0.836 (0.666–1.050)	0.123	1.291 (0.747–2.228)	0.360
LDL-C at 0 months	1.323 (0.873–2.006)	0.187	0.669 (0.251–1.784)	0.422
Weight at 0 months	1.005 (0.992–1.018)	0.457	1.006 (0.977–1.036)	0.687
Triglycerides at 0 months	0.941 (0.750–1.179)	0.595	1.279 (0.672–2.433)	0.453
HbA1c at 12 months	0.801 (0.629–1.020)	0.072	0.834 (0.484–1.439)	0.514
LDL-C at 12 months	1.154 (0.763–1.745)	0.497	1.098 (0.403–2.988)	0.855
Triglycerides at 12 months	0.739 (0.507–1.076)	0.115	0.577 (0.245–1.358)	0.208

*Note:* Non-Indigenous ethnicity is taken to be ethnicity identified as not Aboriginal and/or Torres Strait Islander. Statistically significant results are shown in bold (*p* ≤ 0.05).

Abbreviations: 95% CI, 95% confidence interval; BMI, body mass index; DBP, diastolic blood pressure; DPP4, dipeptidyl-peptidase 4; HbA1c, hemoglobin A1c; LDL-C, low-density lipoprotein cholesterol; OR, odds ratio; SBP, systolic blood pressure; SGLT2, sodium-glucose cotransporter 2.

## Data Availability

The paper contains all the necessary data. Due to privacy and confidentiality concerns, the full underlying dataset cannot be publicly shared. However, anonymized summary data can be made available upon submission of a request and subsequent approval by an appropriate Human Research Ethics Committee, in accordance with data protection regulations.
